# Ruminative thinking mediates the effects of exposure to adverse life events on psychotic-like experiences

**DOI:** 10.3389/fpsyg.2024.1434470

**Published:** 2024-11-12

**Authors:** Leonardo Fazio, Alessandra Raio, Tobias Banaschewski, Arun L. W. Bokde, Sylvane Desrivières, Herta Flor, Hugh Garavan, Penny Gowland, Antoine Grigis, Andreas Heinz, Jean-Luc Martinot, Marie-Laure Paillère Martinot, Eric Artiges, Frauke Nees, Dimitri Papadopoulos Orfanos, Tomáš Paus, Luise Poustka, Michael N. Smolka, Sarah Hohmann, Nathalie Holz, Nilakshi Vaidya, Henrik Walter, Robert Whelan, Gunter Schumann, Alessandro Bertolino, Giulio Pergola, Linda A. Antonucci, The IMAGEN Consortium

**Affiliations:** ^1^Department of Medicine and Surgery, Libera Università Mediterranea (LUM) University “Giuseppe Degennaro”, Bari, Italy; ^2^Department of Translational Biomedicine and Neuroscience, University of Bari Aldo Moro, Bari, Italy; ^3^Department of Child and Adolescent Psychiatry and Psychotherapy, Central Institute of Mental Health, Medical Faculty Mannheim, Heidelberg University, Mannheim, Germany; ^4^German Center for Mental Health (DZPG), Partner Site Mannheim-Heidelberg-Ulm, Mannheim, Germany; ^5^Discipline of Psychiatry, School of Medicine and Trinity College, Institute of Neuroscience, Trinity College Dublin, Dublin, Ireland; ^6^Social Genetic and Developmental Psychiatry Centre, Institute of Psychiatry Psychology and Neuroscience, King's College London, London, United Kingdom; ^7^Institute of Cognitive and Clinical Neuroscience, Central Institute of Mental Health, Medical Faculty Mannheim, Heidelberg University, Mannheim, Germany; ^8^Department of Psychology, School of Social Sciences, University of Mannheim, Mannheim, Germany; ^9^Departments of Psychiatry and Psychology, University of Vermont, Burlington, VT, United States; ^10^Sir Peter Mansfield Imaging Centre, School of Physics and Astronomy, University of Nottingham, Nottingham, United Kingdom; ^11^NeuroSpin Commissariat à l'Energie Atomique et aux Energies Alternatives (CEA), Université Paris-Saclay, Gif-sur-Yvette, France; ^12^Department of Psychiatry and Psychotherapy, CCM Charité—Universitätsmedizin Berlin Corporate Member of Freie Universität Berlin, Humboldt-Universität zu Berlin, Berlin Institute of Health, Berlin, Germany; ^13^Institut National de la Santé et de la Recherce Médicale, INSERM U A10 “Trajectoires développementales & psychiatrie”, University Paris-Saclay, Ecole Normale Supérieure Paris-Saclay, CNRS, Centre Borelli, Gif-sur-Yvette, France; ^14^AP-HP. Sorbonne Université, Department of Child and Adolescent Psychiatry, Pitié-Salpêtrière Hospital, Paris, France; ^15^Psychiatry Department, EPS Barthélémy Durand, Etampes, France; ^16^Institute of Medical Psychology and Medical Sociology, University Medical Center Schleswig Holstein, Kiel University, Kiel, Germany; ^17^Department of Psychiatry and Neuroscience, Faculty of Medicine, Centre Hospitalier Universitaire Sainte-Justine, University of Montreal, Montreal, QC, Canada; ^18^Department of Psychiatry, McGill University, Montreal, QC, Canada; ^19^Department of Child and Adolescent Psychiatry, Center for Psychosocial Medicine, University Hospital Heidelberg, Heidelberg, Germany; ^20^Department of Psychiatry and Psychotherapy, Technische Universität Dresden, Dresden, Germany; ^21^Centre for Population Neuroscience and Stratified Medicine (PONS), Department of Psychiatry and Psychotherapy, Charité Universitätsmedizin Berlin, Berlin, Germany; ^22^School of Psychology, Global Brain Health Institute, Trinity College, Dublin, Ireland; ^23^Centre for Population Neuroscience and Precision Medicine (PONS), Institute for Science and Technology of Brain-inspired Intelligence (ISTBI), Fudan University, Shanghai, China; ^24^Psychiatry Unit, Policlinico di Bari, Bari, Italy; ^25^Lieber Institute for Brain Development, John Hopkins Medical Campus, Baltimore, MD, United States; ^26^Department of Psychiatry and Behavioral Science, John Hopkins University, Baltimore, MD, United States

**Keywords:** rumination, ruminative response, adverse life events, psychotic-like experiences, clinical psychology, psychosis risk

## Abstract

**Introduction:**

A growing literature has shown that exposure to adverse life events during childhood or adolescence is associated with the presence of psychotic-like experiences (PLEs), which is in turn associated with the risk of psychotic outcomes. Ruminative thinking, i.e., the tendency to dwell on particular issues or ideas, may affect the perceived aversiveness and ability to cope with adverse life events. However, the role that rumination plays in the relationship between adverse life events and the presence of PLEs remains unclear. The purpose of this study is to assess the association between adverse life events and PLEs in a longitudinal sample of young adults and adolescents, and to investigate whether this relationship is mediated by ruminative thinking.

**Methods:**

We used a longitudinal naturalistic sample of 706 volunteers assessed at ages 18 and 22 years, within the Imagen consortium. Lifetime occurrence of adverse life events (i.e., events perceived as strongly negative by participants) was investigated using the Life Events Questionnaire. The Community Assessment of Psychic Experience (CAPE-42) served to assess the presence of PLEs, while ruminative thinking was investigated through the Ruminative Response Scale.

**Results:**

Results showed that both frequency of PLEs and their persistence over time were associated with greater adverse life events exposure (*r* = 0.32, *p* < 0.001 and *F*_1_ = 9.8; *p* < 0.001, respectively) and greater ruminative response (*r* = 0.66, *p* < 0.001 and *F*_1_ = 94.9; *p* < 0.001, respectively). Mediation analyses showed that relationship between adverse life events and PLEs frequency was partially mediated by rumination (direct effect Z: 5.4, *p* < 0.001; indirect effect Z: 6.9, *p* < 0.001; total effect Z: 5.9, *p* < 0.001). Considering changes between the two assessment timepoints, relationship between PLEs variation between 18 and 22 years and adverse life events occurred during the same period was partially mediated by changes in rumination (direct effect Z: 2.8, *p* < 0.005; indirect effect Z: 4.3, *p* < 0.001; total effect Z: 4.3; *p* < 0.001).

**Discussion:**

Overall, our findings confirm that the presence of adverse life events may increase the risk of experiencing PLEs in healthy individuals and suggest that dysfunctional coping strategies, such as ruminative thinking, may be related to psychosis proneness. Results do not disentangle whether individuals with greater risk for psychosis tend to ruminate more or whether rumination exacerbates psychosis risk.

## 1 Introduction

Psychotic-like experiences (PLEs) are perceptions, thoughts, or beliefs that are considered strange, unusual, or unreal, but do not meet the clinical threshold for psychotic disorders (Linscott and Van Os, 2013; Mark and Toulopoulou, 2016; Rossler et al., 2007; Staines et al., 2022; Hinterbuchinger and Mossaheb, 2021). They may include hallucinations or delusions in a subclinical form (Staines et al., 2022), as well as the subthreshold presence of negative symptoms, thought disturbances, or affective disorders (Mark and Toulopoulou, 2016). PLEs are common in the general population, especially in childhood and adolescence (Linscott and Van Os, 2013; Mcgrath et al., 2015; Staines et al., 2023), while they tend to decrease in the transition to adulthood (Verdoux et al., 1998; Rubio et al., 2012; Laurens et al., 2012; Staines et al., 2023).

While reported as common experiences in the general population, multiple studies suggest that a more pronounced presence of PLEs in childhood and adolescence may be considered an early marker for later psychiatric vulnerability (Lindgren et al., 2022; Zhang et al., 2019; Yung et al., 2006), and may be associated with a higher risk of onset of different types of mental health disorders (Healy et al., 2019; Isaksson et al., 2020). Many studies indicate that PLEs predict the risk of developing overt psychotic disorders (Zammit et al., 2013; Sullivan et al., 2020). Other studies pointed out that it is the persistence of PLEs over time, rather than their mere presence, that may be associated with a higher risk for psychotic disorders (Calkins et al., 2017; Linscott and Van Os, 2013; Kalman et al., 2019).

Interestingly, recent literature suggested that the exposure to adverse life events may impact both presence and persistence of PLEs, thereby shaping psychotic risk (Trotta et al., 2015; Zhang et al., 2019). Indeed, as extensively documented for psychosis (Varese et al., 2012; Popovic et al., 2019; Misiak et al., 2017; Varchmin et al., 2021), the role played by exposure to adverse life events, especially in childhood and adolescence, is also relevant for PLEs (Mcgrath et al., 2017). Adverse events that seem to be associated both with psychotic symptoms and PLEs include traumatic experiences such as physical maltreatment and abuse, emotional abuse, neglect, and abandonment (Mcgrath et al., 2017; Dhondt et al., 2023; Stickley et al., 2021), as well as individual life experiences perceived as aversive, such as moving to another city, the breakup of a friendship, or economic and social difficulties (Newbury et al., 2023; Wang et al., 2019; Loch et al., 2017; Tan et al., 2021).

Several psychological attributes and resources, such as emotional regulation, experiential avoidance, rumination, appraisal, psychological flexibility and mindfulness (Lincoln et al., 2017; Flouri and Mavroveli, 2013; Fernandez-Fernandez et al., 2020; Browne et al., 2022; Ortiz and Sibinga, 2017; Swanepoel et al., 2020; Boyes et al., 2016; Lovatt et al., 2010), might affect the individual reactivity and the perceived magnitude of stressful events, modulating part of the subjective load related such events, as well as their fallout (Misiak et al., 2017). However, some psychological processes, such as rumination, might influence more prominently and directly the subjective consequences of stressful events.

Rumination is the repetitive, prolonged, and recurrent thinking about oneself, one's feelings, personal concerns, and experiences (Watkins and Roberts, 2020), and may even include judgmental pondering on oneself, one's negative emotional states and unmet standards (Treynor et al., 2003, Takano and Tanno, 2009). Evidence reported that rumination processes can directly shape individual responses to stressful events by exacerbating and prolonging emotional states in response to such events and associated negative thoughts (Lask et al., 2021; Watkins and Roberts, 2020; Nolen-Hoeksema et al., 2008; Padilla Paredes and Calvete Zumalde, 2015; Ciesla et al., 2011). Conversely, also the exposure to stressful events can lead to increased rumination. Indeed, conceptual models on the etiology of rumination suggest that repeated experiences of adverse life events can drive rumination not only about those events but also about many areas of an individual's life (Nolen-Hoeksema et al., 2008; Nolen-Hoeksema, 1991; Conway et al., 2004), contributing to the structuring of a ruminative response trait (Spasojević and Alloy, 2002, Shaw et al., 2019; Nolen-Hoeksema, 1991).

For these reasons, it is not surprising that rumination has been associated with several mental health disorders, such as depression, anxiety or bipolar disorder (Snyder et al., 2019; Kovacs et al., 2020; McLaughlin and Nolen-Hoeksema, 2011; Grierson et al., 2016), and has been considered a transdiagnostic factor of mental health vulnerability (Snyder et al., 2019; Wong et al., 2023; Silveira et al., 2020; Hsu et al., 2015). There is consistent literature on the relationship between rumination and psychosis, indicating a greater presence of rumination in patients with psychosis (Vorontsova et al., 2013; Halari et al., 2009; Thomas et al., 2014), and a positive association between rumination and the presence of delusions in patients with psychosis (Jones and Fernyhough, 2009; Hartley et al., 2014; Freeman et al., 2015).

Despite this evidence, the type of relationship linking rumination and PLEs under naturalistic conditions is still unclear. Importantly, some studies suggest that rumination is crucial in the pathway linking exposure to adverse events and PLEs development. This possibility is based on two levels of evidence: on the one hand, it is well-documented that adverse events can promote rumination in non-clinical individuals (Shaw et al., 2019; Nolen-Hoeksema et al., 2008) especially in adolescence (Shaw et al., 2019). A recent study conducted on adolescents, for example, found that exposure to moderate or high levels of stress related to different aspects of daily life, such as peer relationships or family life, can increase rumination, consolidating a ruminative response style (Shaw et al., 2023). Notably, stressful life events increase rumination levels and are associated with lower psychological wellbeing also in adult samples (Michl et al., 2013). On the other hand, rumination may also elicit transient psychotic symptoms in healthy individuals. For example, studies in non-clinical individuals have shown that the experimental induction of rumination under stressful conditions increases hallucinatory perceptions (Hartley and Morrison, 2017). Other studies show that experimental conditions favoring rumination increase paranoid ideation in healthy young volunteers (Martinelli et al., 2013). In line with this evidence, it has been suggested that rumination may increase the use of intrusive thoughts, which in turn would elicit the onset of nuanced psychotic symptoms (Jones and Fernyhough, 2009). Other authors have proposed that ruminative thinking promotes the maintenance of delusional beliefs, as it may deprive individuals of the cognitive flexibility and mindful self-focus needed to disconfirm them (Simpson et al., 2012, Martinelli et al., 2013; McKie et al., 2017). Taken together, these findings suggest that rumination might mediate the relationship between exposure to adverse events and PLEs, acting as an intermediate key factor in the causal process (MacKinnon and Luecken, 2008) linking adverse events and PLEs.

Based on these assumptions, this study aims to investigate, in a community-based longitudinal study of adolescents and young adults, the contribution of exposure to adverse life events and individual ruminative response to determining the frequency and persistence of PLEs.

We hypothesize that greater exposure to adverse life events would be associated with, on the one hand, higher individuals' ruminative response, and, on the other hand, higher frequency and persistence of PLEs. Furthermore, we expect that the ruminative response may mediate the relationship between exposure to adverse life events and both the frequency of PLEs and their persistence over time.

## 2 Materials and methods

### 2.1 Participant selection

Participants were selected from the IMAGEN study dataset (Mascarell Maricic et al., 2020; Schumann et al., 2010). The IMAGEN study is a multisite, multinational longitudinal project that was carried out in eight European sites in England, Ireland, France, and Germany. The study involved a total cohort of more than 2000 14-year-old adolescents recruited from high schools. To obtain a diverse sample in terms of socio-economic status, emotional and cognitive development, private, state-funded, and special units have been equally targeted.

All participants underwent four waves of assessment for different data domains (biological samples, brain imaging, clinical characteristics, and functioning data). These assessments were conducted at baseline (14 years of age), and then followed up at 16, 19, and 22 years (i.e., follow-up 1, follow-up 2, and follow-up 3, respectively).

Exclusion criteria included: the presence of overt neurological conditions such as epilepsy, brain tumors, bacterial infections of the CNS, muscular or myotonic dystrophy; cerebral trauma with loss of consciousness of more than 30 min; developmental issues such as major neurodevelopmental disorders, nutrition and metabolic diseases, uncorrectable visual or auditory deficits, IQ < 70; treatment for schizophrenia or bipolar disorder; presence of medical condition such as type 1 diabetes, systemic rheumatologic disorders, malignant tumors requiring chemotherapy, congenital heart defects or cardiac surgery, aneurysms; pre/perinatal issues such as maternal diabetes during pregnancy, excessive alcohol use of the mother during pregnancy, premature birth < 35 weeks and/or detached placental, hyperbilirubinemia requiring transfusion; MRI contraindication such as the presence of metal or electronic implants and severe claustrophobia. A detailed description of recruitment and research procedures has been published elsewhere (Schumann et al., 2010).

Clinical and behavioral assessments were performed using Psytools software (Delosis Ltd, London, UK) via its Internet-based platform. The battery of questionnaires and cognitive tasks was self-administered both at participants' homes and at neuroimaging facilities. Participants and their parents provided informed consent, and ethics committees of all participating institutions approved the study.

For the present study, we included the time points at which variables related to exposure to adverse life events, presence of PLEs, and ruminative response were recorded, i.e., the second and third follow-ups (19 and 22 years, respectively). Participants who had a complete assessment of all these three variables at both timepoints were then selected, resulting in a sample of 706 participants (288 M, 418 F).

### 2.2 Psychological and life event assessments

#### 2.2.1 Assessment of adverse life events

Life events were assessed retrospectively with the Life Events Questionnaire (LEQ), self-administered online. This multidimensional questionnaire, which has been validated in adolescents (Newcomb and Harlow, 1986), allows for screening of 39 different life events. For each type of event, both desirability and occurrence are noted. Desirability is detected by labeling each life event among those listed as positive, negative, or neutral on a 5-point scale (from−2, strongly negative, to +2, strongly positive), regardless of its actual occurrence. Occurrence, on the other hand, investigates whether each event actually takes place in the time interval between the previous time point and the current assessment. Because we were specifically interested in adverse life events exposure, we calculated the frequency of occurrence of events with negative desirability (-2 or−1) for each participant. For this study, we used both the total frequency (LEQ-total—which includes events experienced between the initial assessment and the third follow-up) and the frequency recorded in the most recent follow-up (LEQ-recent—which includes events experienced between the second and third follow-up).

#### 2.2.2 Assessment of ruminative response

Scores from the Ruminative Response Scale (RRS; Nolen-Hoeksema, 1991) were considered for the assessment of individual ruminative response. The RRS is a self-report questionnaire based on response style theory (Nolen-Hoeksema, 1991) and includes 22 items, scored on a 4-point Likert scale (from almost never to almost always). The total score (RRS-total) can be calculated by summing the scores of all 22 items. All scales were assessed at both the second and third follow-ups. We also calculated the difference in ruminative response between the second and third follow-up (RRS-total-diff), so that larger values indicate an increase in ruminative response between the two follow-ups.

#### 2.2.3 Assessment of PLEs

The Community Assessment of Psychic Experiences (CAPE), in its 42-item version, was used to assess PLEs (Stefanis et al., 2002). The CAPE is a self-report measure that assesses PLEs across the life course and consists of 42 items that include positive symptoms (20 items), negative symptoms (14 items), and depressive symptoms (eight items). Responses for each item are recorded on a 4-point Likert scale from 1 to 4 indicating frequency and distress. For this study, we used the sum of the symptomatology frequency score (CAPE-freq). We did not include measures of distress as they were potentially related to the rumination dimension (Watkins and Roberts, 2020; Zoccola and Dickerson, 2012). The CAPE scale was administered at the second and third follow-ups. We calculated an indicator (CAPE-freq-diff) of the change in PLEs between the second and third follow-ups, specifically the difference between the scores at the third follow-up and the scores at the second follow-up, such that larger values indicate an increase in PLEs between the two follow-ups. Finally, we calculated a categorical variable (CAPE-persistence), representing the presence or absence of a decrease in PLEs symptomatology between the two follow-ups, which should better capture the clinical marker PLEs persistence with adulthood (Calkins et al., 2017).

### 2.3 Statistical analysis

The variables of interest were checked for the potential presence of outliers using Grubb's test (*p* < 0.05). We adopted the criteria of skewness < 2 and kurtosis < 7 to identify deviations from normality (Kim, 2013), and we found that all variables had acceptable normality. Finally, to assess the potential effect of confounding factors, we tested the association between demographic variables (age and sex) and the variables of interest using Pearson's correlations and Student's *t*-tests (*p* < 0.05). The effect of sex was significant for most of the variables considered (see below), so sex was included as a covariate in subsequent analyses. Descriptive statistics are shown in Table 1.

**Table 1:** The descriptive analyses of the sample.

***N***			**AGE**	**RRS-total**	**CAPE-freq**		**LEQ-total**
706	Follow-up 2	*Mean*	18.4	37.6	19.9	*Mean*	10
		*SD*	0.6	11.6	11	*SD*	4.8
♂ 284	*SEX (T-test)*			T−4.1^***^	T−3^**^		T−5.4^***^
♀417	*AGE (Pearson's r)*			n.s.	n.s.		n.s.
							LEQ-recent	
	Follow-up 3	*Mean*	22	38.9	17.4	*Mean*	2.2
		*SD*	0.6	12	10.4	*SD*	1.8
	*SEX (T-test)*			T-4.7^***^	n.s.		T−2^*^
	*AGE (Pearson's r)*			n.s.	n.s.		n.s.

We provided the mean and standard deviation values for the main variables, separately for follow-up 2 and follow-up 3. We reported the results of the association statistics of the main variables with sex (by Student's t) and age (by Pearson's correlation).

RRS-total, Ruminative Response Scale, total score; CAPE-freq, Community Assessment of Psychotic Experience, frequency of the psychotic-like experience; LEQ-total, Life Events Questionnaire, total score; LEQ-recent, Life Events Questionnaire, score related to the exposure to recent adverse events.

^*^p < 0.05; ^**^p < 0.01; ^***^p < 0.001.

Statistical analyses were conducted using JASP 0.18 software for Mac (https://jasp-stats.org/).

To investigate the relationship between lifetime exposure to adverse life events and the presence of PLEs, we performed Pearson's correlation analysis (*p* < 0.05) between LEQ-total and CAPE-freq scores. Also, with Pearson's correlation (*p* < 0.05), we explored the relationship between lifetime exposure to adverse life events (LEQ-total) and the ruminative response (RRS-total). Finally, Pearson's correlation (*p* < 0.05) served to assess the relationship between CAPE-freq and the RRS-total. All correlations were corrected for multiple comparisons using the false discovery rate procedure [Benjamini-Hochberg method (Benjamini, 1995)]. To test the role of the variables LEQ- total and RRS-total in determining CAPE-freq levels, we calculated a regression model (*p* < 0.05) by including CAPE-freq as the dependent variable, and LEQ- total and RRS-total as predictors and sex as a covariate.

We conducted longitudinal analyses to assess the selective effect of exposure to adverse life events between follow-up 2 and follow-up 3 on the presence of PLEs and ruminative response. Specifically, we performed a Pearson's correlation (*p* < 0.05) between LEQ-recent and CAPE-freq-diff, as well as between LEQ-recent and the RRS-total-diff. Again, correlations were corrected for multiple comparisons using the false discovery rate procedure (Benjamini, 1995). To test the role of the LEQ-recent and RRS-total-diff in determining the score of CAPE-freq-diff, we calculated a regression model (*p* < 0.05) by including CAPE-freq-diff as the dependent variable, LEQ-recent and RRS-total-diff as predictors and sex as a covariate.

We employed ANOVA models [*p* < 0.05, with Benjamini-Hochberg correction (Benjamini, 1995)] to compare individuals who exhibited persistence of PLEs between follow-ups 2 and 3 with those who reported a reduction in the same time frame. This was done to ascertain whether the clinical marker of PLEs persistence (Calkins et al., 2017) was associated with exposure to adverse life events (both lifetime and between follow-ups 2 and 3). Specifically, we performed an ANOVA with LEQ-total score as the dependent variable and CAPE-persistence and sex as categorical predictors. Another ANOVA model was calculated using the LEQ-recent score as the dependent variable and CAPE-persistence and sex as categorical predictors. We also investigated the relationship between ruminative response changes and persistence of PLEs frequency by calculating an ANOVA model [*p* < 0.05, with Benjamini-Hochberg correction (Benjamini, 1995)] with RRS-total-diff as the dependent variable and CAPE-persistence and sex as categorical predictors.

The potential significant associations between adverse life events, PLEs, and ruminative responses were further analyzed with different mediation analysis models, aiming to explore the potential mediating role of ruminative response (Baron and Kenny, 1986).

Specifically, to investigate the role of ruminative response on the relationship between lifetime adverse life events exposure and PLEs frequency at follow-up 3, we created a mediation model with the LEQ-total measure as the predictor, CAPE-freq scores as the outcome, RRS-total as the mediator, and sex as the covariate. An additional mediation analysis was performed to explore the role of ruminative response on the relationship between adverse life events exposure between follow-up 2 and follow-up 3 and changes in PLEs frequency over the same period. Specifically, we computed a mediation model with LEQ-recent as the predictor, CAPE-freq-diff as the outcome, sex as the covariate, and RRS-total-diff, as the mediator. All the models were bootstrapped for 1,000 repetitions, with significance set at p < 0.05.

We conducted additional statistical analyses to better detail the association between specific types of life events (e.g., family/parenting events, accident/illness events, sexual events, autonomy events, deviance events, relocation events, and distress events) and the various dimensions of PLEs.

Finally, to ensure that our results were not affected by sample size reduction due to the stringent sample selection process based on the selection of complete cases, we replicated the analyses using a multiple imputation method for missing data. The statistics and results of these analyses are presented in the Supplementary material.

## 3 Results

Analyses conducted to test for the potential effect of confounding factors did not show an association between age and the variables of interest. There was a clear sex effect, with female participants reporting higher levels of adverse life events (LEQ-total: t-5.4 *p* < 0.001; LEQ-recent: t-2 *p* = 0.04), a greater ruminative response [RRS-total (follow-up2): t-4.1 *p* < 0.001; RRS-total (follow-up3): t-4.7 *p* < 0.001], and a greater presence of PLEs at follow-up2 (t-2.9 *p* = 0.004) than male participants.

Correlation analyses between lifetime exposure to adverse life events, presence of PLEs, and ruminative response showed a positive association between LEQ-total and CAPE-freq scores (*r* = 0.32, *p* < 0.001), as well as a positive association between LEQ-total and RRS-total scores (*r* = 0.27, *p* < 0.001). We also found positive correlations between CAPE-freq and RRS-total scores (*r* = 0.66, *p* < 0.001). Regression shows that LEQ-total and RRS-total scores significantly predict CAPE-freq scores [*F*_(3, 704)_ = 199.4, *p* < 0.001], with a significant effect of both RRS-total (Beta = 0.63; *t* = 21.4; *p* < 0.001) and LEQ-total (Beta = 0.16; *t* = 5.4; *p* < 0.001).

Correlation analyses carried out on the scores calculated using data from both follow-ups, showed a positive association between LEQ-recent and CAPE-freq-diff (*r* = 0.16, *p* < 0.001), as well as between LEQ-recent and RRS-total-diff score (*r* = 0.13; *p* < 0.001). We also found positive correlations between the CAPE-freq-diff score and the RRS-total-diff score (*r* = 0.47; *p* < 0.001). Regression on the scores calculated using data from both follow-ups shows that LEQ-recent and RRS-total-diff scores significantly predict CAPE-freq-diff scores [*F*_(3, 704)_ = 71.5, *p* < 0.001], with a significant effect of both RRS-total-diff (Beta = 0.46, *t* = 13.7; *p* < 0.001) and LEQ-recent (Beta = 0.1, *t* = 3; *p* = 0.002).

ANOVAs revealed a higher number of adverse life events between follow-up 2 and follow-up 3 among participants who had increased PLEs frequency during the same period, compared with those who had not increased it (CAPE-persistence - LEQ-recent: *F* = 9.8; df = 1; *p* < 0.001). In contrast, the two groups showed no difference in the number of adverse life events experienced in their lifetime (CAPE-persistence - LEQ-total – *p* > 0.05). Both ANOVAs showed no interaction between CAPE-persistence and sex (*p* > 0.05). Similarly, ANOVA revealed a greater increase in ruminative response between follow-up 2 and follow-up 3 among participants who had increased PLEs frequency during the same period, compared with those who had not (CAPE-persistence - RRS-total: *F* = 94.9; df = 1; *p* < 0.001). In addition, we found an interaction between CAPE-persistence and sex (CAPE-persistence^*^sex- RRS-total F = 6.6; df = 1; *p* = 0.01), indicative of a more pronounced ruminative response increase in female subjects.

**Figure 1:**
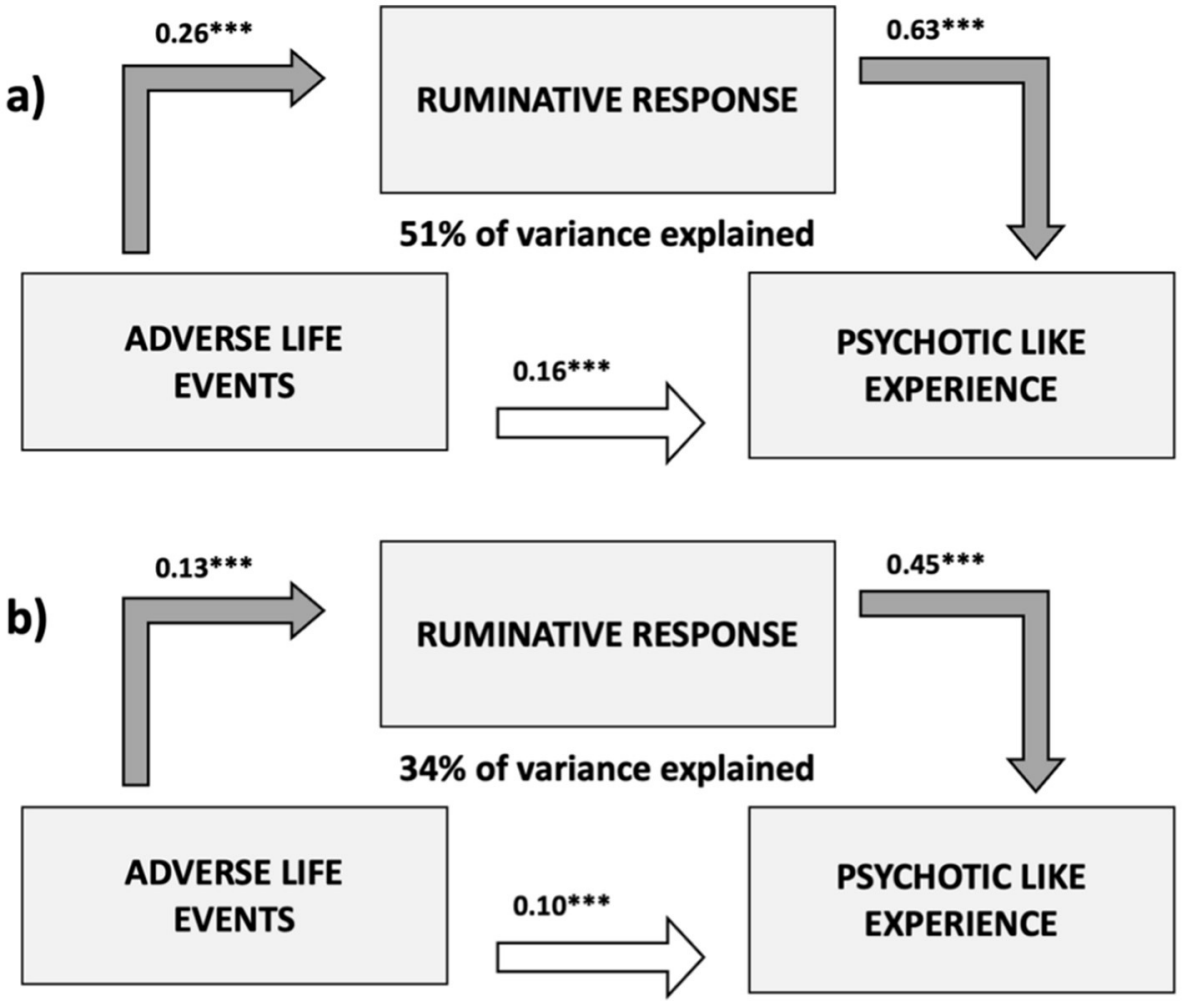
Graphical representation of the mediating role of ruminative response on the relationship between exposure to adverse life events and frequency of psychotic-like experiences. **(a)** Depicts the results for the measurements taken at follow-up 3. **(b)** Depicts the results for the scores calculated as the difference between follow-up 2 and follow-up 3. The values above each arrow indicate the standardized path coefficient estimates. *p < 0.05; **p < 0.01; ***p < 0.001.

Mediation analyses were conducted on the variables that showed a significant association between adverse life events, PLEs, and ruminative response, according to Baron and Kenny (1986). We found partial mediation of RRS-total on the relationship between LEQ-total and CAPE-freq (direct effect - estimate 0.158, 95% Confidence Interval - C.I.-.: 0.093–0.224, Z: 5.4, *p* < 0.001; indirect effect - estimate 0.168, 95% C.I.: 0.118–0.219, Z: 6.9, *p* < 0.001- 51% of total effect explained; total effect - estimate 0.326, 95% C.I.: 0.248–0.413, Z: 8.9, *p* < 0.001, Figure 1a).

Similarly, we found a partial mediation of changes at the RRS scale between follow-up 2 and follow-up 3 (RRS-total-diff) on the relationship between LEQ-recent and CAPE-freq-diff (direct effect - estimate 0.163, 95% C.I.: 0.103–0.218, Z: 2.8, *p* = 0.005; indirect effect - estimate 0.163, 95% C.I.: 0.103–0.218, Z: 4.3, *p* < 0.001- 34% of total effect explained; total effect - estimate 0.163, 95% C.I.: 0.103–0.218, Z: 4.3, *p* < 0.001, Figure 1b).

## 4 Discussion

In this study, we investigated the association between exposure to adverse life events and PLEs in a community-based sample of adolescents and young adults, as well as the role of the ruminative response in mediating this relationship. Our results support the hypothesis that both lifetime and recent exposures to adverse life events are associated with a higher occurrence of PLEs in adolescents and young adults. Furthermore, our results indicate that the relationship between aversive life events and PLEs is mediated by the recourse individuals make to ruminative thinking, potentially indicating rumination as a mechanism through which exposure to aversive life events may determine the onset and persistence of PLEs, conditioning the associated risk of developing psychosis.

More in detail, our results show the existence of a positive association between exposure to adverse life events and PLEs. We found a greater occurrence of PLEs, in particular, in individuals who were exposed to more events experienced (subjectively) as adverse in their lives. This finding reflects fully previous work reporting more PLEs in individuals exposed to adverse life events, especially in childhood or adolescence (Varese et al., 2012; Trotta et al., 2015; Morgan and Gayer-Anderson, 2016; Mcgrath et al., 2017). Furthermore, our results indicate that the association between PLEs frequency and exposure to adverse events persists even when we consider only recent adverse life events exposure, i.e., related to the last 2 years. These results suggest the possibility that more recent aversive or acute distress conditions may also increase the present occurrence of PLEs (Grant and Hennig, 2020; Cristobal-Narvaez et al., 2016), even if the lifetime occurrence of such events is not considered.

The investigation of the relationship between recent exposure to adverse life events and present PLEs occurrence is relevant, as it could affect the persistence of PLEs over time. Our analyses indicated that subjects who reported a persistent frequency of PLEs between the second and third follow-ups were more likely to have been exposed to recent adverse events than those who reported a reduced frequency of PLEs. However, there was no difference between the two groups in terms of lifetime adverse event exposure. Overall, these results support the hypothesis of a specific contribution of recent stressors in conditioning the current occurrence of PLEs (Cristobal-Narvaez et al., 2016). However, the evaluation of these results must take into account the retrospective modalities we used to record events, which may have made the detection of the occurrence of remote adverse events less accurate, magnifying the impact of the more recent events.

In exploring the relationship between exposure to adverse life events and PLEs, we then focused on the role played by mental rumination. We found a positive association between ruminative response and adverse events, and in particular a greater ruminative response in the presence of greater lifetime exposure to adverse events, as well as in the presence of greater recent exposure.

This result is consistent with previous studies, as rumination has been associated not only with adverse events during childhood or adolescence (Mansueto et al., 2021; Munoz and Hanks, 2021; Conway et al., 2004) but also with current stressors (LeMoult et al., 2013) although, as noted above, this finding may parallel the greater impact of adverse events in our survey. Alternatively, exposure to adverse events during an individual's development may contribute to structuring a stable ruminative response in the individual (Shaw et al., 2019; Just and Alloy, 1997). On the other hand, our results are reminiscent of other studies, describing rumination as a state sensitive to current stressors (LeMoult et al., 2013). These two dimensions are interconnected, as evidence shows that frequent exposure to adverse events may not only increase rumination about these events but also consolidate a pattern of ruminative response to adversity, which in turn can magnify the associated distress (Nolen-Hoeksema, 1991; Spasojević and Alloy, 2002, Shaw et al., 2019).

In our sample, in addition, the ruminative response appeared positively associated with PLEs, indicating a higher frequency of PLEs in individuals with a greater ruminative response. In addition, individuals who reported persistence of PLEs between the second and third follow-up also reported a greater increase in ruminative response. These findings tie in with previous evidence showing that, in patients with psychosis, symptomatology may be predicted by a greater tendency to ruminate (Jones and Fernyhough, 2009; Hartley et al., 2014; Freeman et al., 2015), as well as previous work on non-clinical populations that, under experimental conditions, associates ruminative thinking with paranoid ideation (Martinelli et al., 2013; McKie et al., 2017).

The association between ruminative responses with exposure to adverse life events and PLEs was confirmed through regression analysis. The findings indicate that both adverse life events and ruminative responses play a significant role in the frequency of PLEs and changes in PLEs over time. The nature of this relationship was clarified by mediation analyses, which revealed that rumination acts as a partial mediator between adverse life events and PLEs. Given the positive association of rumination with the two aforementioned dimensions, it is, therefore, possible that rumination acts as an amplifier of the detrimental effect of exposure to adverse life events, increasing the possibility that the individual will experience PLEs. Although there are no data describing this mechanism of PLEs, studies of patients with psychosis report a relationship between rumination and symptomatology in the presence of adverse life events or trauma (Fang et al., 2023; Chung et al., 2021; Liu et al., 2020; Ludwig et al., 2020), supporting the plausibility of this mediating mechanism. This would suggest that part of the PLEs experienced by individuals can be attributed to mechanisms that associate the experience of adverse events with a strengthening of the ruminative response, potentially through amplification of distress conditions that would increase PLEs (Ludwig et al., 2019; Collip et al., 2013; Kelleher et al., 2015). Additionally, we find that even focusing on the most recent events, i.e., those occurring between the second and third follow up, the relationship between the frequency of adverse events and the increase in PLEs over the same period is partially mediated by changes in the ruminative response. The ruminative response is thus confirmed as a process sensitive to stressor exposure that is, in turn, capable of enhancing distress conditions, potentially promoting emotional dysregulation (Lask et al., 2021; Watkins and Roberts, 2020; Nolen-Hoeksema et al., 2008; Padilla Paredes and Calvete Zumalde, 2015; Ciesla et al., 2011) with implications for the possibility of experiencing PLEs (Bak et al., 2005; Kramer et al., 2012; Collip et al., 2013; Kelleher et al., 2015).

Our work highlighted the role of rumination processes in the relationship between exposure to adverse events and the occurrence of psychotic-like symptomatology in a “naturalistic” population. The role of exposure to adverse life events in critical periods, such as childhood and adolescence has been widely described for several conditions of mental health impairment, particularly psychosis (Varese et al., 2012). The trajectories linking adverse events to the outcome of psychosis, however, are unclear, as they converge on the interplay of environmental, genetic, physiological, and psychological factors (Misiak et al., 2017). The results of our work highlight the role of a specific potentially critical psychological process among these factors, namely ruminative responses. We can postulate that rumination is bidirectionally related to stressor exposure: on one hand, it is influenced by exposure to stressors, becoming more dysfunctional in repeated exposure to stressors (Watkins and Roberts, 2020; Nolen-Hoeksema, 1991). On the other hand, frequent recourse to rumination processes may be capable of amplifying the magnitude of these same events by enhancing their associated distress (Conway et al., 2004; Nolen-Hoeksema et al., 2008), with potentially critical effects on the individual's mental health. This may be a mechanism not only for amplifying but also for chronicizing the effects due to exposure to adverse life events.

Furthermore, we investigated subclinical experiences of psychosis rather than considering the diagnosis of psychosis. This approach is advantageous in that it allows us to investigate more nuanced symptomatic conditions, in which it may be easier to see small effects due to psychological variables such as ruminative processes. This also may be the appropriate field for testing the relationships between these factors and other modulators of the risk of developing psychosis, such as genetic or other environmental factors. Moreover, focusing on PLEs allows us to investigate the psychological mechanisms underlying the development of psychotic illness net of the alterations in thoughts that may be common in individuals with overt psychosis, as well as net of the effects of drug therapy. Last, this approach is most appropriate for investigation in adolescence or young adulthood, as it potentially anticipates transitions to psychosis.

Our work also has limitations. A first limitation may be the use of measures of exposure to adverse events based solely on retrospective self-reports. Although this mode of investigation is common, given the retrospective nature of this information and despite the substantial reliability of such measures (Aalsma et al., 2002; Bernstein et al., 1994; Fink et al., 1995; Riddle and Aponte, 1999), the use of retrospective self-reports can raise doubts regarding the actual occurrence of reported episodes (McKinney et al., 2009) or the assessment of the subjective impact of adverse events (Danese and Widom, 2020; Francis et al., 2023). A second limitation is the absence of a replication sample. Although our study involves a large number of individuals and a longitudinal measurement, replications of the results in an external sample would have allowed us to validate the results obtained, and the robustness of the statistical design adopted, increasing the scientific relevance of our work (Laws, 2016). A final substantial limitation is that changes in ruminative response may be an effect rather than a cause of increased PLEs, or that in the presence of high levels of rumination or a high frequency of PLEs, the recall of past events is experienced as more aversive. Further studies are warranted to test this hypothesis.

In addition to these limitations, other issues deserve attention and will need to be investigated in the future. The main variables considered in our work show a relevant effect of sex. This is not unexpected, as these associations have already been described in the literature (Johnson and Whisman, 2013; Stainton et al., 2021; Kajantie and Phillips, 2006; Kendler et al., 2001; Kudielka and Kirschbaum, 2005). However, it is important to note that the relationship between our variables of interest and sex may influence the mechanisms by which they are associated. For example, there is evidence that women report a greater number of adverse events (Kendler et al., 2001), and this may be related to several factors, including a different physiological response to stressors (Kajantie and Phillips, 2006; Kudielka and Kirschbaum, 2005), different behavioral response patterns (Taylor et al., 2000), but also a different use of coping strategies with the adverse experience, such as the ruminative response (Liu et al., 2023; Shull et al., 2016; Staugaard and Berntsen, 2021). The combination of these factors (Shull et al., 2016) may influence the trajectories that lead to psychological outcomes (Kendler et al., 2001), potentially increasing the risk of PLEs in women.

Another point to consider is the socio-cultural composition of our sample. Indeed, the data analyzed in this paper were collected in different European countries and may not be generalizable to other cultures or social contexts. Several papers report cultural differences in the way people respond to traumatic or negative experiences and in the use of the ruminative response. In particular, individualistic Western cultures use rumination less than Eastern cultures (De Vaus et al., 2018; Nisbett et al., 2001), but in the latter rumination is less maladaptive and less associated with psychological outcomes. Similarly, the prevalence and importance of PLEs vary by ethnic background and country of origin (Lewis-Fernandez et al., 2023), suggesting that the data reported in this paper may be limited to the socio-cultural context analyzed. Further cross-cultural research is therefore needed to investigate how the variables examined may interact in different contexts.

Despite these limitations, our results, which highlight a modulatory role of ruminative thinking in the relationship between adverse life experiences and PLEs, have the potential to inform clinical practice. In light of these findings, it can be postulated that therapeutic interventions aimed at reducing the use of ruminative thinking (Querstret and Cropley, 2013) may prove an effective strategy in reducing PLEs, helping to contain the risk of developing psychosis, particularly in individuals exposed to adverse life events. In line with studies that proposed therapeutic interventions based on modifying metacognitive processes (Giugliano et al., 2022; Moritz et al., 2019), with a particular focus on rumination, at different stages of psychosis (Hutton et al., 2014; Balzan et al., 2019; Clemmensen et al., 2024; Moritz et al., 2019), our results suggest that such interventions could also be effective in conditions preceding psychosis, with a potential effect on the frequency of PLEs. Moreover, such interventions could be even more effective in individuals exposed to adverse life events, in whom a reduced reliance on ruminative thought processes could reduce the psychological outcomes due to exposure to such events. Therefore, future studies will have to investigate how additional environmental, genetic, or physiological factors interact with ruminative processes in triggering psychotic-like experiences, and how to develop effective psychological interventions aimed at reducing the reliance on rumination, thus helping to contain the risk of developing psychosis.

## Data Availability

The access to the dataset used in this study is regulated by the IMAGEN Consortium (https://imagen-project.org/). Requests to access these datasets should be directed to andreas.heinz@charite.de.
