# Supplementary material for: Ruminative thinking mediates the effects of exposure to adverse life events on psychotic-like experiences
Source: Front Psychol. 2024 Nov 12;15:1434470. doi: 10.3389/fpsyg.2024.1434470 (PMC11589823; doi:10.3389/fpsyg.2024.1434470)
Supplement: Supplementary file 1 [file Data_Sheet_1.PDF]

Supplementary Material

The supplementary material presented here describes (i) the consort chart that graphically represents the process of selecting the study sample from the IMAGEN dataset, (ii) a replication of the study results using a larger sample obtained from the imputation of missing data on the IMAGEN dataset, and (iii) some exploratory analyses conducted to investigate specific relationships between different types of adverse life events and different dimensions of PLE. These analyses can be used to develop specific hypotheses that can be tested in future studies.

1. Consort Chart depicting the study sample selection

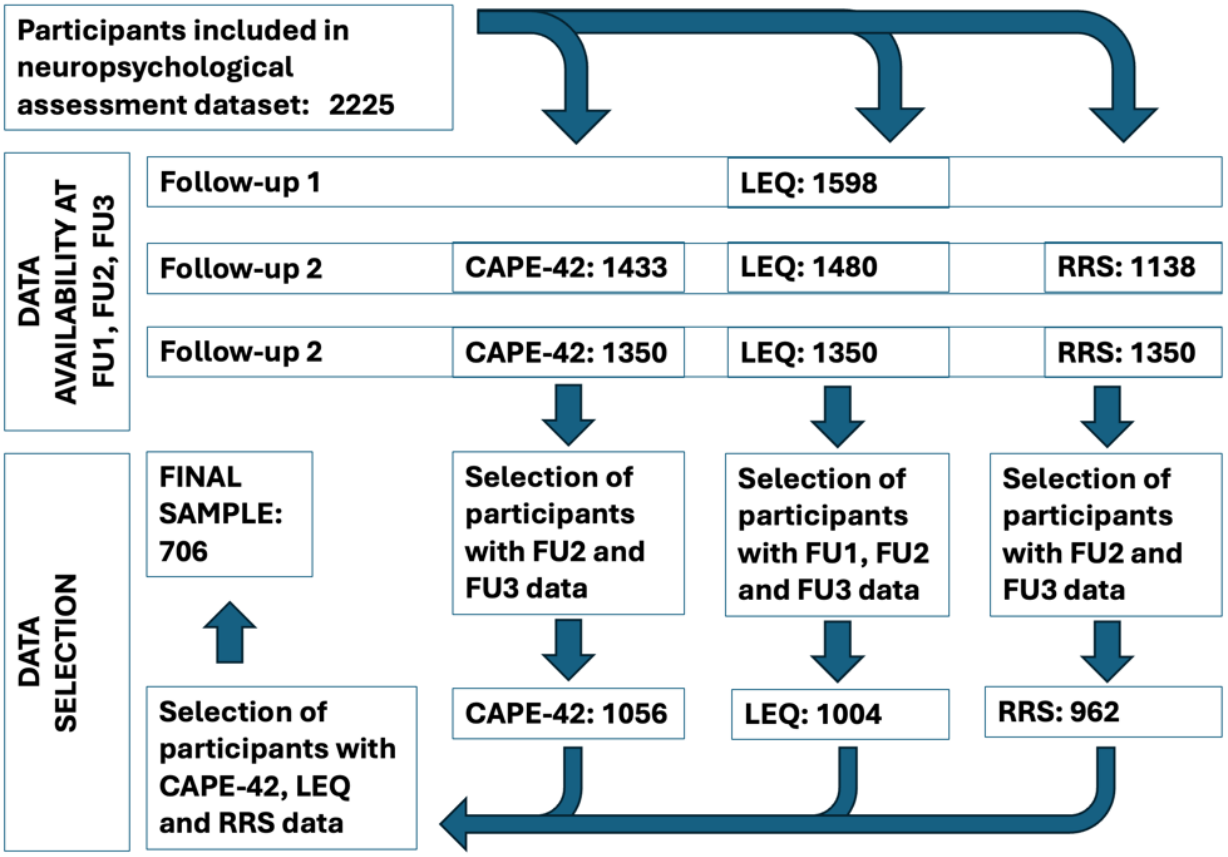

Abbreviations. RRS: Ruminative Response Scale; CAPE-42: Communitiy Assessmet of Psychotic Experience; LEQ: Life Events Questionnaire, FU1: follow-up 1, FU2: follow-up 2, FU3: follow-up 3

2. Replication of the study results using multiple imputation

To ensure that our results were not affected by sample size reduction due to the stringent sample selection process based on the selection of complete cases, we replicated the analyses using a multiple imputation method for missing data.

We started with the sample of 1598 people for whom we had at least data available in follow-up 1, thus excluding only participants who had been exclusively recorded during baseline. This allowed us to contain the use of imputation, which range between 1% to 29%, depending on the variables Multiple imputation was performed with the R package "mice" (cran.r-project.org/web/packages/mice/index.html), with the following parameters: method = Predictive Mean Matching, number of iterations = 50, seed = 500)

Correlation findings obtained through imputed data replicated those obtained on non-imputed data. We found a positive association between LEQ-total and CAPE-freq scores ($r=0.32$, $p<0.001$), as well as a positive association between LEQ-total and RRS-total scores ($r=0.18$, $p<0.001$), and a positive correlations between CAPE-freq and RRS-total scores ($r=0.49$, $p<0.001$). We also replicated correlation analyses carried out on the scores calculated using data from both follow-ups, that showed a positive association between LEQ-recent and CAPE-freq-diff ($r=0.13$, $p<0.001$) and between the CAPE-freq-diff score and the RRS-total-diff score ($r=0.39$; $p<0.001$). We however, do not replicated the association between LEQ-recent and RRS-total-diff score ($r=0.04$; $p=0.13$).

Regression findings obtained through imputed data fully replicated those obtained on non-imputed data. We found that that LEQ-total and RRS-total scores significantly predicted CAPE-freq scores ($F_{3,1595} = 221.2$, $p<0.001$), with a significant effect of both RRS-total (Beta = 0.45 $t = 20.4$; $p<0.001$) and LEQ-total (Beta = 0.25 $t = 11.2$; $p<0.001$). Similarly, we found that LEQ-recent and RRS-total-diff scores significantly predict CAPE-freq-diff scores ($F_{3,1595} = 101.2$, $p<0.001$), with a significant effect of both RRS-total-diff (Beta = 0.38, $t =16.3$; $p<0.001$) and LEQ-recent (Beta = 0.12, $t = 4.9$; $p<0.001$).

ANOVA models performed on imputed data fully replicated those obtained on non-imputed data. In detail, we found a higher number of adverse life events between follow-up 2 and follow-up 3 among participants who had increased PLEs frequency during the same period, compared with those who had not increased it (CAPE-persistence - LEQ-recent: $F=27$; $df=1$; $p<0.001$). In contrast, the two groups showed no difference in the number of adverse life events experienced in their lifetime (CAPE-persistence - LEQ-total - $p>0.05$). Both ANOVAs showed no interaction between CAPE-persistence and sex ($p>0.05$). Similarly, ANOVA revealed a greater increase in ruminative response between follow-up 2 and follow-up 3 among participants who had increased PLEs frequency during the same period, compared with those who had not (CAPE-persistence - RRS-total: $F=140.2$; $df=1$; $p<0.001$). In addition, we found an interaction between CAPE-persistence and sex (CAPE-persistence*sex- RRS-total $F=5.9$; $df=1$; $p=0.02$), indicative of a more pronounced ruminative response increase in female subjects.

Finally, we replicated the mediation analyses investigating the mediation of RRS-total on the relationship between LEQ-total and CAPE-freq, finding a partial mediation (direct effect - estimate 0.25, 95% Confidence Interval - C.I.-.: 0.201-0.296, Z: 11.2, $p<0.001$; indirect effect - estimate 0.08,

95% C.I.: 0.054-0.107, Z: 6.6, p<0.001; total effect - estimate 0.329, 95% C.I.: 0.274-0.377, Z: 13.3, p<0.001). However, we do not replicate the mediation of changes at the RRS scale between follow-up 2 and follow-up 3 (RRS-total-diff) on the relationship between LEQ-recent and CAPE-freq-diff.

Overall, findings on imputed data showed a substantial overlap with findings obtained on non-imputed data, with only two exceptions pertaining to (i) the correlation between LEQ-recent and RRS-total-diff score, and (ii) the mediation analysis of the RRS-total-diff score on the relationship between LEQ-recent and CAPE-freq-diff. It should be noted that both analyses involved the use of longitudinal indices derived from score differences between follow ups 2 and 3, and therefore the lack of replication might have been affected by the use of non-real, imputed data, especially considering that the percentage of imputed data for the computation of the RRS-total-diff score was considerable (42%).

3. Exploratory analyses on specific relationships between different types of adverse life events and different dimensions of PLE

3.1 Assessment of life events and PLEs

The LEQ instrument (Newcomb, 1986) allows for the differentiation of the various life events under consideration by categorizing them into seven distinct categories indicating respectively, family/parenting events (FAM), accident/illness events (ACC), sexual events (SEX), autonomy events (AUT), deviance events (DEV), relocation events (REL), and distress events (DIS). For each event, we calculated the frequency of events with negative desirability (score -2 or -1) for each participant. We then calculated the total frequency relative to each dimension, obtaining the LEQ-FAM-total, LEQ-ACC-total, LEQ-SEX-total, LEQ-AUT-total, LEQ-DEV-total, LEQ-REL-total and LEQ-DIS-total scores, respectively. Similarly, we calculated the frequency of such events recorded in the most recent follow-up (LEQ-recent-which includes events experienced between the second and third follow-ups), resulting in LEQ-FAM-recent, LEQ-ACC-recent, LEQ-SEX-recent, LEQ-AUT-recent, LEQ-DEV-recent, LEQ-REL-recent, and LEQ-DIS-recent scores.

The CAPE-42 (Stefanis et al., 2002) allows for the differentiation of positive, negative, and depressive components of LEQs. Specifically, the CAPE-42 has 18 items of positive symptoms, 14 of negative symptoms, and 8 of depressive symptoms. Responses for each item are recorded on a 4-point Likert scale from 1 to 4 indicating frequency and distress. For the following supplementary analyses, we used the sum of symptom frequency scores for the positive (CAPE-P-freq), negative (CAPE-N-freq), and depressive (CAPE-D-freq) dimensions of PLEs. The CAPE scale was administered at the second and third follow-ups. We calculated indicators of the change in PLEs size between the second and third follow-ups (CAPE-P-freq-diff, CAPE-N-freq-diff, CAPE-D-freq-diff), specifically the difference between the scores at the third follow-up and the scores at the second follow-up, such that larger values indicate an increase in PLEs between the two follow-ups.

3.2 Statistical Analysis

To investigate the specific relationships between the different types of adverse life events and the different dimensions of PLEs, we replicated some of the analyses performed in the main study, but differentiated both by the categories of adverse events reported in the LEQ and by the different dimensions of PLEs reported in CAPE-42. To test the associations between the variables considered,

we performed Pearson's correlation analysis ($p<0.05$) by including the total frequency scores at all event categories recorded on the LEQ scale and the total scores in each of the CAPE-42 dimensions, namely CAPE-P-freq, CAPE-N-freq and CAPE-D-freq. Similarly, we investigated by Pearson's correlation analysis ($p<0.05$) the association between the frequency scores of adverse events recorded in the third follow-up, in each of the categories in the LEQ, and the changes to the CAPE-42 scores between the second and third follow-ups, namely CAPE-P-freq-diff, CAPE-N-freq-diff and CAPE-D-freq-diff. All correlations were corrected for multiple comparisons using the false discovery rate procedure (Benjamini-Hochberg method (Benjamini, 1995)).

To test the contribution of ruminative response in mediating the relationship between specific categories of adverse events and specific dimensions of PLEs, we conducted mediation analyses considering LEQ variables as predictors, ruminative response as mediator, and CAPE-42 dimensions as outcomes, similar to what was done in the main study. Specifically, we included in the mediations the variables in which a correlation emerged between predictor and outcome (Baron and Kenny, 1986) (see below).

We then performed a mediation analysis by including as predictors the variables LEQ-FAM-total, LEQ-SEX-total, LEQ-AUT-total, LEQ-DEV-total, and LEQ-DIS-total, as mediator the RRS-total scores, as outcome the CAPE-P-freq, and as covariate the sex. A further mediation analysis was performed by including as predictors the variables LEQ-FAM-total, LEQ-SEX-total, and LEQ-DIS-total, as mediator the RRS-total scores, as outcome the CAPE-F-freq, and as covariate the sex. Finally, a mediation analysis was performed by including as predictors the variables LEQ-FAM-total, LEQ-SEX-total, LEQ-DEV-total, LEQ-REL-total, and LEQ-DIS-total, as mediator the RRS-total scores, as outcome the CAPE-D-freq, and as covariate the sex. All the models were bootstrapped for 1,000 repetitions, with significance set at $p<0.05$.

In addition, we performed mediation analyses to assess the contribution of the change in ruminative response between follow-up 2 and follow-up 3 in mediating the relationship between the adverse experience measured at follow-up 3, in the different event categories and the changes in CAPE-42 scores between follow-up 2 and follow-up 3. Also for this analysis, we included in the mediations the variables in which a correlation emerged between predictor and outcome (Baron and Kenny, 1986) (see below). Specifically, a mediation analysis was carried out by including as predictors the LEQ-DIS-recent variables, as mediator the RRS-total-diff scores, as outcome the CAPE-F-freq-diff, and as covariate the sex. Another mediation analysis was performed by including as predictors the variables LEQ-FAM-recent and LEQ-DIS-recent, as mediator the RRS-total-diff scores, as outcome the CAPE-D-freq-diff, and as covariate the sex. All the models were bootstrapped for 1,000 repetitions, with significance set at $p<0.05$.

3.3 Results

Correlation analyses show a positive association of the CAPE-P-freq dimension with LEQ-FAM-total (r 0.18, $p>0.001$), LEQ-SEX-total (r 0.2, $p>0.001$), LEQ-AUT-total (r 0.11, $p=0.003$), LEQ-DEV-total (r 0.14, $p>0.001$) and LEQ-DIS-total (r 0.23, $p>0.001$) scores. The CAPE-F-freq dimension shows a positive association with LEQ-FAM-total (r 0.17, $p>0.001$), LEQ-SEX-total (r 0.16, $p>0.001$, and LEQ-DIS-total (r 0.27, $p>0.001$) scores. The CAPE-D-freq dimension shows a positive association with LEQ-FAM-total (r 0.19, $p>0.001$), LEQ-SEX-total (r 0.25, $p>0.001$), LEQ-DEV-total (r 0.12, $p=0.001$), LEQ-REL-total (r 0.11, $p=0.004$) and LEQ-DIS-total (r 0.35, $p>0.001$) scores. In addition, there was a positive association of the CAPE-F-freq-diff dimension with LEQ-

DIS-recent scores (r 0.13 p>0.001), and a positive association of the CAPE-D-freq-diff dimension with LEQ-FAM-recent (r 0.13 p>0.001) and LEQ-DIS-recent (r 0.13 p>0.001) scores

Looking at the mediation analysis, in the model aimed at investigating the mediating role of ruminative response on the relationship between different categories of adverse events and the positive dimension of PLEs, we found a complete mediation of rumination on the relationship between the variables LEQ-FAM-total- CAPE-P-freq (direct effect Z: 1.9, p=0. 6; indirect effect Z: 3.1, p=0.002; total effect Z: 2.8, p=0.005), and LEQ-DIS-total- CAPE-P-freq (direct effect Z: 1.9, p=0.6; indirect effect Z: 4.9, p<0.001; total effect Z: 3. 7, p<0.001) and partial mediation for the LEQ-SEX-total- CAPE-P-freq relationship (direct effect Z: 2.5, p=0.01; indirect effect Z: 2.6, p=0.01; total effect Z: 3.2, p=0.001).

In the model aimed at investigating the mediating role of ruminative response on the relationship between different categories of adverse events and the negative dimension of PLEs, we found a complete mediation of rumination on the relationship between the variables LEQ-FAM-total- CAPE-F-freq (direct effect Z: 5.4, p=0. 8; indirect effect Z: 3.3, p<0.001; total effect Z: 2, p=0.04) and LEQ-SEX-total- CAPE-F-freq (direct effect Z: 0.8, p=0.3; indirect effect Z: 2.7, p=0.008; total effect Z: 2. 2, p=0.03) a partial mediation for the LEQ-DIS-total - CAPE-F-freq relationship (direct effect Z: 2.5, p=0.01; indirect effect Z: 6.1, p<0.001; total effect Z: 5.7, p<0.001)

In the model aimed at investigating the mediating role of ruminative response on the relationship between different categories of adverse events and the depressive dimension of PLEs, we found partial mediation of rumination on the relationship between the variables LEQ-SEX-total- CAPE-D-freq (direct effect Z: 3. 5, p<0.001; indirect effect Z: 2.7, p=0.007; total effect Z: 4.6, p<0.001) and LEQ-DIS-total- CAPE-D-freq (direct effect Z: 4, p<0.001; indirect effect Z: 6.2, p<0.001; total effect Z: 7.3, p<0.001).

Mediation analyses that investigated changes in scores between follow-up 2 and follow-up 3 reported partial mediation of ruminative response variation (RRS-total-diff) on the relationship between the variables LEQ-DIST-total-diff- CAPE-D-freq-diff (direct effect Z: 2. 4, p=0.016; indirect effect Z: 2.9, p=0.004; total effect Z: 3.4, p<0.001), as well as on the relationship between the variables LEQ-DIST-total-diff- CAPE-D-freq-diff (direct effect Z: 2.8, p=0.004; indirect effect Z: 2.6, p=0.01; total effect Z: 3.8, p<0.001).
